# Memorable media messages of mental illness and implications for policy support: examining the influences of racial ingroup/outgroup recall

**DOI:** 10.3389/fpsyg.2025.1568155

**Published:** 2025-07-23

**Authors:** Julius Matthew Riles, Abigail Adediran, Esther Akheituame, Guadalupe Madrigal, Elizabeth Behm-Morawitz

**Affiliations:** ^1^Department of Communication, University of Missouri, Columbia, SC, United States; ^2^Department of Communication, University of California, Santa Barbara, Santa Barbara, CA, United States

**Keywords:** media, memorable messages, mental health, policy support, social identity, race

## Abstract

**Introduction:**

It is vital to understand how memorable media representations of mental illness influence perceptions about, and policies affecting, people managing mental illness.

**Method:**

Utilizing an experiment, this study empirically examines how ingroup/outgroup racial identity recall of those managing mental health conditions may condition respondents' perceptions of mental illness more broadly, including support for allocating mental health resources.

**Results:**

Findings suggest that the ingroup/outgroup racial identity recall of a memorable media message significantly predicts broader culpability judgments of people managing mental illness and support for mental health-related policies. Unexpectedly, memorable media message recall of racial outgroups was associated with less perceived culpability for a mental illness than recall of racial ingroups. However, content analysis of the recalled messages reveals that, among other cues, explicit emphasis of race, negative language, and perceived potential for messages to influence audiences were most pronounced during recall of racial outgroups with mental illnesses than recall of racial ingroups.

**Discussion:**

Implications of patterns for health disparity support for mental illness, as well as mediated memorable message research are discussed.

## Introduction

The experiences of mental illness (MI) are ubiquitous. In the United States, one of five adults are currently experiencing a mental health condition [Centers for Disease Control and Prevention (CDC), 2024]. There are more than 200 mental health illnesses recognized in the U.S., with the most common ones being anxiety disorder, depression, bipolar disorder, and post-traumatic stress disorder. Despite its prevalence, evidence suggests that public perceptions of MI are pervasively stigmatized (Quintero Johnson and Riles, 2018; Stuart, 2006). The images that media consumers receive about MI from popular message sources are correspondingly associated with stigmatizing disparagement and blame (Riles et al., 2021a). For example, Diefenbach and West's (2007) content analysis of characters with a MI revealed that such characters are 10 times more often portrayed as violent than characters without a MI. Similarly, in an examination of 30 years of popular film, Riles et al. (2021b) observed that characters with MI were disproportionately linked with aggression (physical, verbal, and sexual), as well as a diminished ability to effectively manage adversity, relative to characters without MI. Quintero Johnson and Riles (2018) summed up prior media portrayal studies by noting that “stereotypic depictions of MI are characterized by an emphasis on the violent, dangerous, unpredictable, and childlike nature of people who are perceived to have a MI, the presentation of the most severe illnesses and illness symptoms, and the use of derogatory and demeaning labels to signify MI” (p. 148). As such, many public images of MI suggest a threat, though less is clear regarding the content of audience's memories of these depictions.

The majority of the studies evaluating the influences of mediated MI portrayals have examined outcomes of exposure situations (e.g., Kresovich, 2022; Lee et al., 2021). However, an examination of how particularly salient and memorable prior message experiences could facilitate perceptual and dispositional outcomes toward those with MI is a step that has yet to be concertedly addressed in the literature. This is to say, though research predicting and analyzing influences of media exposure on MI perceptions certainly has scholarly value, how media users recall those media message encounters, in conjunction with the social identities recalled as intersecting with the MI health identity, would logically play a role in determining the salience of various additional schematic attributions (e.g., culpability perceptions).

Media exposure to those managing health conditions influences how individuals are oriented toward supporting those managing these health encounters (Riles et al., 2023), including policy-related support (Smith, 2012). As such, it is logically conceivable that the composition of accessible memorable messages would also contribute to such policy support outcomes as a function of the schematic attributions made salient. However, to our knowledge, no such examination exists. Investigating how media users' recollection of accessible depictions of MI influences subsequent dispositions toward this health identity provides an increasingly robust understanding of how media exposure (including its processing and retention) could influence beliefs and sociopolitical inclinations toward MI. By further examining how racial and ethnic identity contribute to these beliefs and inclinations, notably when intersecting with a MI health identity, we can obtain an increasingly nuanced picture of how intersectional identities create unique health policy preferences. When those recalled racial and ethnic identities are perceived as ingroups, relative to outgroups, such considerations may be expected to contextualize the amount of favorability and support media users are inclined to assign to those managing MI, more broadly.

The current study responds to the aforementioned research gap by examining how the recall of memorable messages of mediated MI portrayals influences desires to be supportive of those managing MI via the backing of health policy. Such patterns are examined within the context of ingroup/outgroup racial identity intersections of the individual recalled to be managing MI, specifically as a function of how those identity intersections yield varying stigmatizing attributions of culpability. This research provides insights to better understand how cognitive retention of media messages about MI can activate potential health disparities in terms of social concern and desires for institutional support of MI.

### Memorable messages of social reality

Research has demonstrated the notable capacity for media exposure to influence how people feel about health and racial identities when they are experiencing hardship. For instance, related to health identity, Riles et al. (2015) observed that common media frames of cancer influence news audiences' perception of those managing cancer. Specifically, consuming cancer news with higher lifestyle-focused framing of illness triggered thoughts of cancer patients as responsible for their hardships and diminished compassion for victims. Related to ethnic identity, Hsueh et al. (2015) observed that exposure to prejudiced comments about Asian international students influenced participants' tendency to post prejudicial comments, themselves, which fed into their conscious and unconscious attitudes toward this identity when offline. In both cases, it was exposure to a particular identity-based depiction that was subsequently associated with diminished support for those individuals, presumably as a function of how those messages influenced cognitive representations of those identities held by media consumers. In addition to examining the initial effects of message exposure on dispositions toward marginalized identities, research must further explore how recall of particularly accessible message experiences could inform various types of affective responses and support inclinations oriented toward those managing social and health concerns (e.g., Smith, 2012). Individuals are routinely bombarded with myriad information in their everyday experiences. However, only a subset of those experiences may resonate as impactful and, therefore, “pulled forward” in uncertain moments of sensemaking and decision-making (Horstman et al., 2024). Memorable messages refer to “distinct communication units considered influential over the course of a person's life, and which draw our attention toward features like source, channel, the relationship between the sender and receiver, and characteristics surrounding reception and enactment” (Cooke-Jackson and Rubinsky, 2023, p. 2). As a socialization technique, memorable messages are (a) recalled for an extended period; (b) have a lasting impact on the individual, whether constructive or destructive; (c) play an important role in an individual's socialization, impacting identity and behavior; and (d) are constituted as salient by their prominent influence (Jordan, 2023, p. 2). These messages can have several evaluative implications for how individuals form impressions regarding various social phenomena, as well as one another. Though often examined within real-world social contexts (e.g., family or other interpersonal sources of message experiences), researchers in these domains note that “media messages function similarly [to interpersonally originating messages] and thus should be considered a potential source of memorable messages” (Horstman et al., 2024, p. 3).

Kerr et al. (2020), in their analysis of memorable messages parents encountered regarding their children's vascular birthmark conditions, found that most of these messages were perceived unfavorably, which had negative implications on their identity as parents. These messages reinforced guilt and confirmed parents' internal fear, producing distress and stigmatizing outcomes related to children such as avoidance and social isolation. This is to say, particularly salient health information recall had notable implications for self-worth and comfort regarding the management of the health situation within social interaction. Greenwell (2019) similarly observed that memorable messages about MI, notably within families, were associated with less positive attitudes toward mental health help seeking, as well as more personal stigma and negative health and relational outcomes. Flood-Grady et al. (2021) correspondingly observed the capacity of parental memorable messages about depression to influence stigma in young adults regarding depression and those diagnosed with it. Though not examining memorable messages originating from the media environment, taken together, this research indicates a prominent role that salient message recall plays in informing broader social and health considerations.

Though not always articulated as such, memorable message effects logic has long existed within theoretical frameworks of media exposure and media effects. The theory of cultivation, for instance, posits that media engagement and viewing contribute to our shared social reality. In the original conception of cultivation, Gerbner et al. (1986) argued that not only can media distort our cultural and social realities, but that this shaped reality aligns much more with our media viewing. Moreover, in terms of media effects from a media psychology approach, theories of heuristics and priming suggest that media messages can activate mental constructs and this memory-based accessibility can influence subsequent ideas, attitudes, and in extreme cases, behaviors (Domke et al., 1998). Finally, in terms of social learning, research also finds that media has the power to influence human thought, affect, and action based on the learned ideas and associations made increasingly accessible in memory (Bandura, 2001). In all, these theories illuminate how media exposure can, and often does, make messages memorable, influencing later message salience.

Despite the ubiquity of media availability, mediated memorable messages have not been extensively explored in terms of their social and health consequences. In one notable exception, Smith et al. (2009), observed that, relative to familial, healthcare, and friend-based sources of information, memorable mediated messages about breast cancer were consistently among the top promoters of ideas pertaining to awareness, prevention, treatment. This is to say, across the lifespan of ways that illness can be encountered, media may be playing a particularly prominent source role in shaping how health views are fixed in memory. Additional research is necessary to explore the social and policy-based implications of such recall, especially when particular social identities are recalled in association with a given health identity. The current study bridges this gap as we center media as a source of memorable messages, exploring the aforementioned implications of recalled identity.

### Memorable media exposure, culpability, and health policy support disparities

Social identity theory (SIT) addresses the contextual implications of intergroup dynamics on potentially biased judgments about the social world (Tajfel and Turner, 1979). This framework suggests that individuals tend to perceptually and behaviorally favor members of their perceived ingroups rather than outgroup members, in part, to secure an affirmative self-concept and achieve positive distinctiveness (Trepte and Loy, 2017). This theory has been used to argue that individuals predominantly interact with others as members of their groups “where one's idiosyncratic, individualizing qualities are overwhelmed by the salience of one's group memberships” (Hornsey, 2008, p. 206). This is to say, people are often evaluated and understood in terms of group memberships that are salient in a given context.

Little to no research has explored the social identity-based implications of ingroup/outgroup memorable message recall on perceptions of, and support for, those recalled. In one rare exception, Soliz et al. (2023) examined memorable messages from families regarding mass suffering (e.g., genocides, natural disasters, etc.) experienced by global outgroups and how such recall was linked with concern for the suffering. These researchers observed that, though some discussion about tangible ways to support outgroups was recalled, far more prevalent were memorable messages rationalizing away the suffering of outgroups (as something that is divinely originating and is less likely to affect the ingroup) and curiosity to comprehend the circumstances of those experiencing suffering (as opposed to concrete support intentions).

Within a mediated context, researchers have observed a tendency for identities associated with the cultural majority to receive more favorable and sympathetic coverage (Dixon, 2019). This is to say, messages featuring different groups, even when they are depicted in parallel contexts or situations, are known to convey different information about those groups. Popular media exposure to a range of minority groups has been associated with increasingly negative views of those groups, including sexual minorities (Fuochi et al., 2020), racial minorities (Arendt et al., 2015; Mastro et al., 2007) and health minorities (e.g., Quintero Johnson and Riles, 2018). Taken together, these studies support the notion that media exposure to popular depictions of societal outgroups (i.e., minorities) is relatively associated with marginalizing influences on perceptions of those groups. It is often argued that such outcomes notably function by way of the schematic activation of culpability for perceptually unfavorable traits (Hooper and Erdogan, 2015). Indeed, according to the fundamental attribution bias framework (Hooper and Erdogan, 2015; Krull et al., 1999), individuals have a general tendency to explain away perceptually negative attributes associated with an ingroup as rooted in less culpable situational reasons (i.e., the circumstances are to blame), whereas similar attributes are more often explained with relatively more culpable dispositional reasons (i.e., the individual is to blame) when associated with outgroups. Evidence suggests that MI is similarly associated with disparate associations with threat and culpability as a function of the social and racial identity of those managing the condition. For example, research has observed that racial and ethnic minorities are often presented as more personally to blame for behaviors associated with mental disorders (Duxbury et al., 2018).

Furthermore, research suggests that ingroup members who perceive outgroup members as associated with a stigmatizing trait are less inclined to support policy related to the outgroup. For instance, studies have revealed that stigmatizing perceptions of both Mexicans and Muslims by American research participants were associated with support for hostile policies targeted toward these groups (Dunwoody and Plane, 2019; Saleem et al., 2016). Indeed, Atwell Seate and Mastro (2016) found that exposure to news about the cultural outgroup of immigrants, particularly when linked with threat, negatively influenced attitudes toward immigrants and indirectly enhanced hostile immigration policy perceptions via intergroup anxiety. Mastro and Kopacz (2006) assert that “the impact of the media on policy decisions is better understood as a…reasoning chain rather than a direct predictor of policy opinions” (p. 319). This is to say, the mediated tendency to recall particular media messages and stereotypes contributes to policy inclinations by way of a chain of reasoning whereby often biased schematic attributions of threat and deservingness, such as those made salient by media exposure, are used in judgments. Hence, it is not just about what viewers are exposed to, but also about the attributional associations they recall and remember, that can presumably influence policy opinions. When individuals saliently recall media messages that reinforce negative stereotypes, they are likely to use these internalized perceptions in broader subsequent judgments.

Taken together, this research suggests that recall of a racial outgroup associated with a stereotypically threatening health identity (i.e., mental illness) would precipitate enhanced perceptions that the individual is culpable for this association, especially relative to recall of a racial ingroup associated with said health identity. Moreover, the degree of culpability perceived in relation to holding the MI identity should predict the magnitude of inclinations to support policy meant to aid those managing MI. This pattern is suggestive of an indirect influence of media portrayals on policy support through viewers' culpability evaluations. Specifically, recalling a message featuring an outgroup may trigger marginalizing stereotype activation, such as stigma culpability judgments, resulting in an influence on policy preferences. As such, we offer the following hypotheses:

H1: Salient recall of a message with a racial outgroup managing MI will be associated with greater culpability perceptions than salient recall of a racial ingroup managing MI.
H2: Greater culpability perceptions will be negatively associated with support for policies meant to aid those with MI.
H3: Recalling a message with an outgroup will be indirectly and negatively associated with policy support, mediated by culpability perceptions.

### Contextualizing role of race in memorable health messages

As aforementioned, there is value in examining the disparate influences of memorable message recall as it relates to ingroup/outgroup status. However, a relatively more nuanced understanding of how health identity recall can influence health perceptions and support inclinations requires an examination of how particular social identities could intersect with health identity in unique ways. In this way, it is necessary to adopt an intersectional identity approach, recognizing that social identities often intersect to potentially compound different forms of marginalization (Jackson et al., 2016). Recently, scholars like Slaughter-Acey et al. (2023) have challenged the common perception that single-axis social identity theorizing could effectively be utilized to make sense of a given individual's health experiences. To this end, there has been advocacy for focusing on multiple marginalized populations and the disparities that may be overlooked when emphasizing only one social category (Carliner et al., 2014). Such calls for increasingly sophisticated social identity treatment have similarly been growing with the field of mediated communication (Ramasubramanian and Banjo, 2020; Riles et al., 2022).

There is a critical need to explore the unique schematic attributes assigned to marginalized populations with MI within an intersectional framework in order to bring greater awareness to mental health disparities, as multiple systems of oppression, contribute to the marginalization and stigmatization of minority groups (Liu et al., 2023; Trygg et al., 2019). An intersectional identity approach to health and illness attributions may also heighten attention regarding notable cultural distinctions important for understanding identity-based prototypical expectations about the management of MI. For example, Bolger et al. (2024) observed Black respondents would more often hold religious explanations and solutions for managing an MI health identity compared to White respondents. Such identity-based distinctions regarding the navigation of MI are only revealed via intersectional considerations of racial and health identity.

A general proposition investigated in the current study concerns the degree to which the racial identity with which people with MI are associated in a memory can disproportionately affect broader culpability perceptions attributed to those managing MI. Available evidence suggests that the popular tendency to assign individual blame for MI behaviors and symptomology is particularly pronounced for racial minorities, especially Black people managing MI (e.g., Duxbury et al., 2018). For instance, Heitzeg (2015) assessed coverage of mass shootings and the conditional tendency to attribute such behaviors, routinely linked with MI, to less culpable medical explanations. They observed that when the perpetrators of such events were White, they were more likely to be assigned medical model situational explanations. The medical model (i.e., disordered behavior caused by illness) is often incorporated by describing perpetrators as simply sick people that deserve public concern and assistance. When Black, and other people of color, were involved in similar disordered behaviors, they were more often associated with dispositional explanations, depicted as “irredeemably evil,” and “thugs” (see Smiley and Fakunle, 2016). Similar patterns have been observed with regard to the stigmatization of substance abuse disorder as it relates to Black individuals (Lindsay and Vuolo, 2021). Altogether, such evidence suggests that depictions of intersecting marginalized racial (e.g., Black) and mental health identity (e.g., mental illness), while not widespread, when present, disproportionately lean toward reinforcing stereotypes of personal culpability for various MI attributes, even in health domains (Riles et al., 2024). As such, salient memorable media messages would be expected to yield schematically different views about MI, and those managing these conditions, depending on who is recalled.

Taken together, it is anticipated that memorable message recall about MI will influence MI policy support inclinations, via racialized culpability perceptions. Though no prior study has undertaken such an examination, Kinder and Winter (2001) observed that White Americans who express hostile views of Black Americans were inclined to oppose various racial policy initiatives, including prohibiting discrimination on the job, federal assistance to Blacks, and quotas in college admissions. Moreover, and in relation to health policy, empirical observation reveals that health policy that is disproportionately viewed as benefitting racial minorities, especially Black individuals, tends to receive far less support and implementation than health policy oriented toward White individuals (e.g., Assari, 2018). As such, we offer the following hypotheses:

H4: The effect of ingroup/outgroup recall will be moderated by the race of those recalled such that outgroup recall of a Black individual with MI will be associated with the most perceived personal culpability.
H5: There will be a moderated mediation effect of ingroup/outgroup recall by race on support for policy meant to aid those with MI, via culpability perceptions.

In addition to examining the influences of recalled memorable mediated messages of MI, there is abundant value in exploring the content of those recollections in order to better understand how specific terminological descriptors may vary as a function of ingroup/outgroup status. For example, particular descriptors of MI as well as references to specific media offerings and platforms may differ as a function of who is recalled. Though prior studies do not permit us to make predictions about precisely how the content of these memories may differ, such descriptions remain valuable to explore because they provide context for better understanding why particular social and policy-based influences of recalled messages were observed. As such, we offer the following research questions:

RQ1: How does the content (e.g., evaluative descriptors and media references) of recalled mediated memorable messages of MI differ between those who recalled racial ingroups relative to racial outgroups?
RQ2: How does the content (e.g., evaluative descriptors and media references) of recalled mediated memorable messages of MI resemble one another between those who recalled racial ingroups relative to racial outgroups?

## Methods

### Sample

Participants (*N* = 296) were recruited from the online research panel recruitment service ResearchMatch, a non-profit program funded by the National Institutes of Health that connects researchers with volunteers to take part in health research studies. With respect to racial/ethnic identification, respondents reported being White (87%), Black or African American (7%), Hispanic or Latino (4%), Asian (1%), and Other (1%). In terms of gender identification, respondents reported being female (77%), male (18%), and non-binary/gender fluid (5%).

### Procedure

Participants engaged in an experiment where they were asked to recall memorable media messages featuring MI. In this two-condition experiment, the primary manipulation was that participants were instructed to either recall and write about a memorable media message of someone who is managing MI from *within* (i.e., ingroup) or *outside* (i.e., outgroup) of their primary racial/ethnic identity. It was emphasized that this could be a social media post, a scene from a series, film, online video, a news article, game, etc., and that “a message is memorable if it has stayed with you and has made any lasting impression.” It was stated that they did not need to agree or disagree with this memorable message, but to write about one media message that was the *most* memorable, including details about the message and descriptions of their experience with it. We specified that we would like them to write roughly a paragraph (i.e., several complete sentences).

### Measures

#### Culpability perceptions

In assessing perceived MI culpability, we adapted a measure from Roscoe and Anderson (2019) created to measure the stigma communication cue of responsibility for the stigmatized trait. Example items include, “The onset of mental illness is often due to the life choices of those experiencing it,” and “Those with a mental illness tend to be personally responsible for developing mental illness.” Participants indicated their agreement with responses ranging from Strongly disagree (coded as 1) and Strongly agree (coded as 5; *M* = 1.71, *SD* = 0.69, α = 0.71).

#### Mental illness public policy support

In assessing inclinations for policies to get the public to collectively support those managing MI, we adapted a measure of perceived deservingness of institutional support from Meuleman et al. (2020). In response to the question stem, “To what extent do you think the government should be responsible for the following tasks…” participants responded to items, including “Making sure those experiencing chronic mental illness have retirement support,” and “Making sure there is affordable health care for those experiencing chronic mental illness” Participants indicated their support on a scale ranging from “No responsibility” (coded as 0) to “Full responsibility” (coded as 10; *M* = 8.15, *SD* = 2.13, α = 0.88).

#### Racial recall

In addition to writing roughly a paragraph about their salient memorable message, respondents were asked questions about their recalled experiences, including the primary race/ethnicity of the person managing MI. In terms of accessible racial/ethnic identifications, participants recalled White (55%), Black or African American (23%), Hispanic or Latino (7%), Asian (4%), and Other (10%) characters.

### Computer-assisted textual analysis

RQ1 and RQ2 were analyzed via sentiment and semantic modeling of the textual data using a Large Language Model (LLM) procedure consistent with prior research (Pilny et al., 2024; Tai et al., 2024). Specifically, OpenAI's ChatGPT-4o was employed to conduct sentiment and semantic modeling of the memorable message data to identify patterns in tone and themes. The textual data were preprocessed, i.e., vectorized using CountVectorizer, to prepare the data for analysis, by, for example, excluding stop words (e.g., a, an, are, in). This procedure is akin to traditional natural language processing (NLP) platforms, but ChatGPT-4o has the added advantage of human-like interpretation of language.

For RQ1, the frequency and distribution of sentiment in the open-ended memorable message responses were analyzed using the lexicon-based approach by TextBlob (Loria, 2018), which is an NLP Python library that uses polarity scores from −1.0 to 1.0, and ultimately categorizes textual data as negative, neutral, and positive.

For RQ2, Latent Dirichlet Allocation (LDA) was performed, using the open-source Python library scikit-learn (Pedregosa et al., 2011), to perceive the underlying themes in the ingroup and outgroup memorable message responses. This machine learning technique is a well-established generative probabilistic model for modeling text and providing insights into the underlying meaning and structure of the data (Blei et al., 2003; Toubia et al., 2019; Yoo et al., 2024).

In addition to uncovering themes, the LDA results were used to examine the coherence, or semantic similarity, of themes across the ingroup and outgroup memorable message responses. Specifically, the trained LDA models for the ingroup and outgroup memorable message responses were analyzed for topic distribution by using Python to identify the topic probabilities and the predominant topic for each model. A multi-layered qualitative analysis was then conducted to assess the coherence of themes across the ingroup and outgroup models. First, we evaluated the themes that emerged from the LDA topic modeling. Second, ChatGPT-4o was prompted to interpret topic coherence and thematic overlap between the ingroup and outgroup models. Third, as researchers, we qualitatively assessed the LLM-generated interpretation of coherence in relation to the original data, focusing on the similarities and differences between the topic models.

## Results

**Figure 1:**
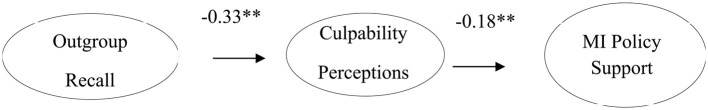
Observed mediation analysis predicting MI policy support via culpability perceptions. Recall of an ingroup serves as referent category. **p < 0.01.

Our first hypothesis predicted that memorable messages of outgroups with MI will be associated with greater culpability perceptions than ingroups. Findings revealed a significant effect of group status [*F*_(1,261)_ = 9.97, *p* < 0.01, η^2^ = 0.04], such that outgroups (*M* = 1.56, *SE* = 0.06) were associated with significantly less culpability perceptions than ingroups (*M* = 1.83, *SE* = 0.05). Thus, findings ran counter to H1 expectations. With regard to H2, predicting that culpability perceptions will be negatively related to policy support, OLS regression results reveal the model to be significant [*F*_(1,266)_ = 12.22, *p* < 0.001, *R*^2^ = 0.19], along with an observed negative relationship (*b* = −0.17, *p* < 0.01, Δ*R*^2^ = 0.02), supporting the hypothesis. With regard to H3, predicting a negative indirect effect of group status on policy support, mediation analyses were conducted using Model 4 of Hayes' (2018) *Process* macro for IBM SPSS statistical software. A positive indirect effect of recalling a message via culpability was observed [*b* = 0.06, 95% *CI* (0.005, 0.134)], running counter to H3. Direct paths can be located in Figure 1.

**Figure 2:**
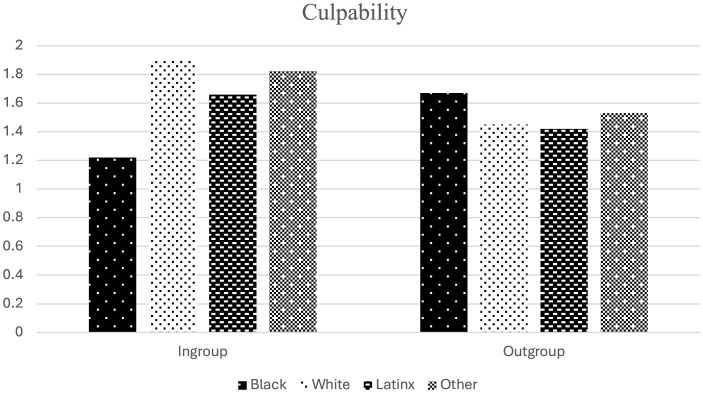
Observed means of culpability perceptions as a function of group status and racial recall.

With regard to H4, predicting an interaction effect of group status and racial recall on culpability perceptions, findings revealed a significant interaction effect of group status and racial recall [*F*_(3,266)_ = 2.96, *p* < 0.05, η^2^ = 0.03]. Decomposition of the interaction reveals that, among those engaged in outgroup recall, relative to recall of Black figures, recall of White figures and the Other group were both significantly associated with diminished culpability perceptions [*b* = −1.41, *p* < 0.05; *b* = −1.10, *p* < 0.05, respectively], while recall of Latinx figures was associated with marginally diminished culpability perceptions [*b* = −1.18, *p* < 0.1]. As such, evidence largely supports H4. See Figure 2 for means.

**Figure 3:**
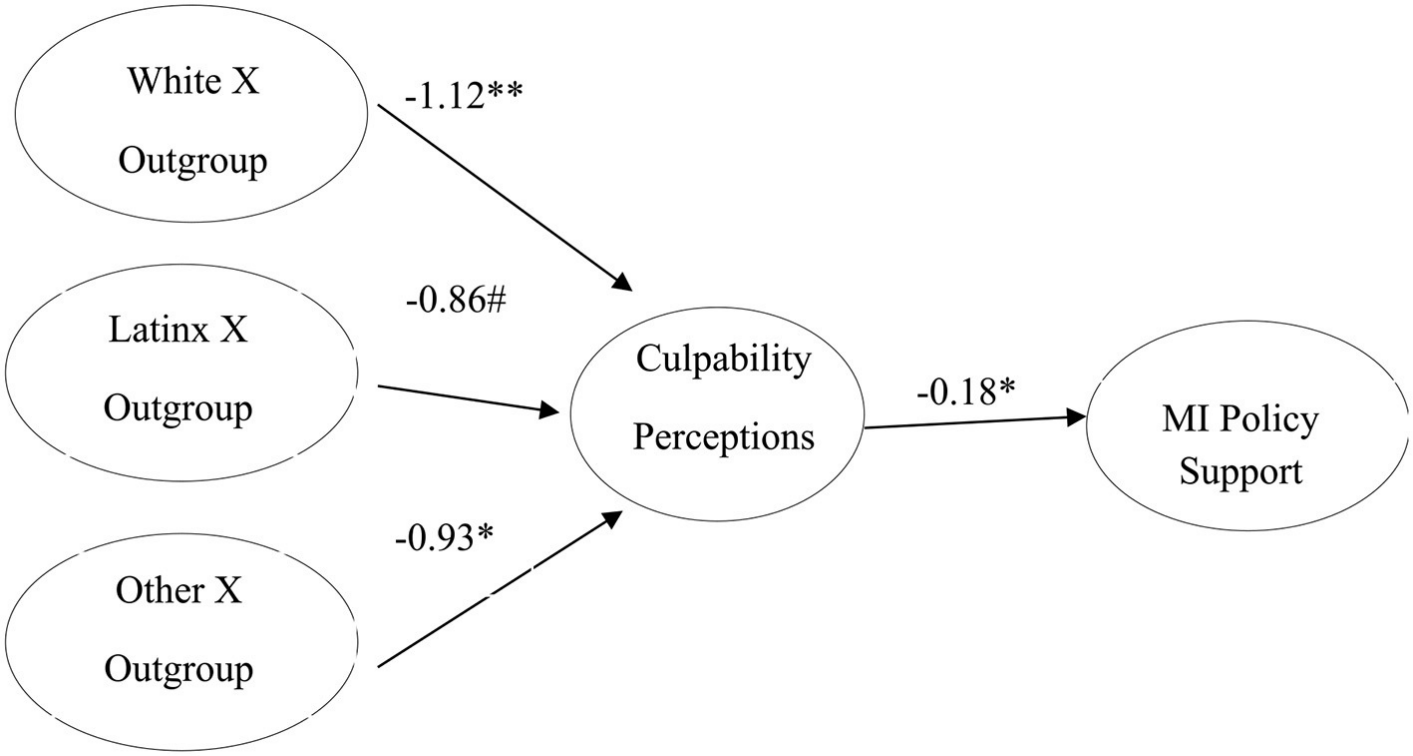
Observed moderated mediation analysis predicting MI policy support via culpability perceptions. Recall of Black characters and ingroups both serve as referent categories. *p < 0.05; **p < 0.01; ^#^*p* < 0.10.

With regard to H5, predicting a moderated mediation effect of group status and racial recall on policy support via culpability perceptions, mediation analyses were conducted using Model 7 of Hayes' (2018) *Process* macro for IBM SPSS statistical software. Findings revealed a significant moderated mediation effect [*b* = *0.0*9, 95% *CI* (0.01, 0.23)], notably regarding ingroup relative to outgroup recall in conjunction with Black relative to White figure recall. Specifically, within those reporting on an ingroup, White respondents reported less support for MI policy than Black respondents (associated with the most ingroup support), and this pattern was mediated by culpability perceptions [*b* = −0.14, 95% *CI* (−0.346, −0.004)]. Furthermore, for those recalling a White character, the recall of an outgroup White character was associated with more support for MI policy than those recalling an ingroup White character, and this pattern was mediated by culpability perceptions [*b* = *0.1*9, 95% *CI* (0.01, 0.46)]. Thus, H5 was supported. Direct paths for this moderated mediation analysis can be found in Figure 3.

**Figure 4:**
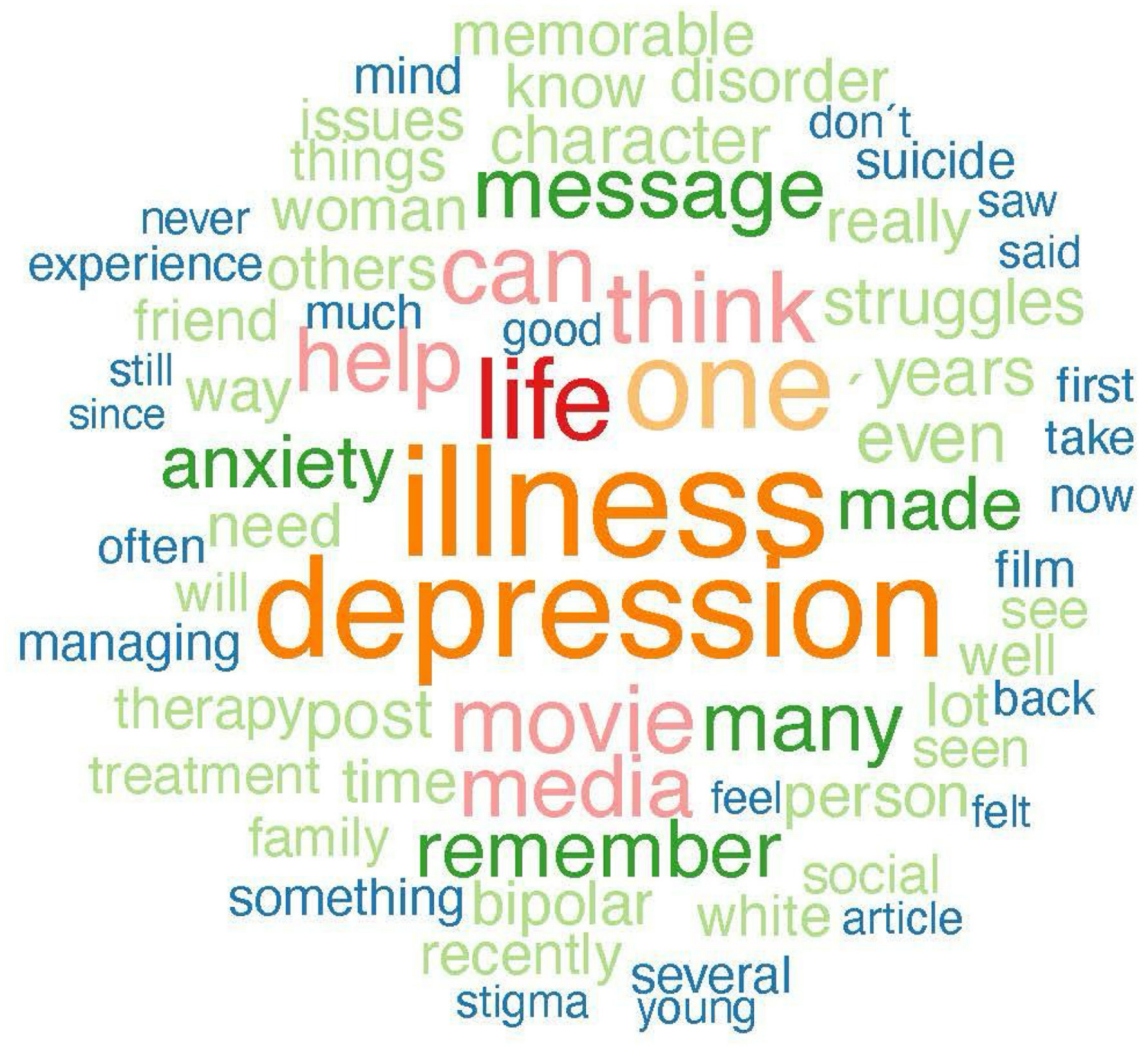
” Ingroup memorable mediated MI message Word Cloud.

**Figure 5:**
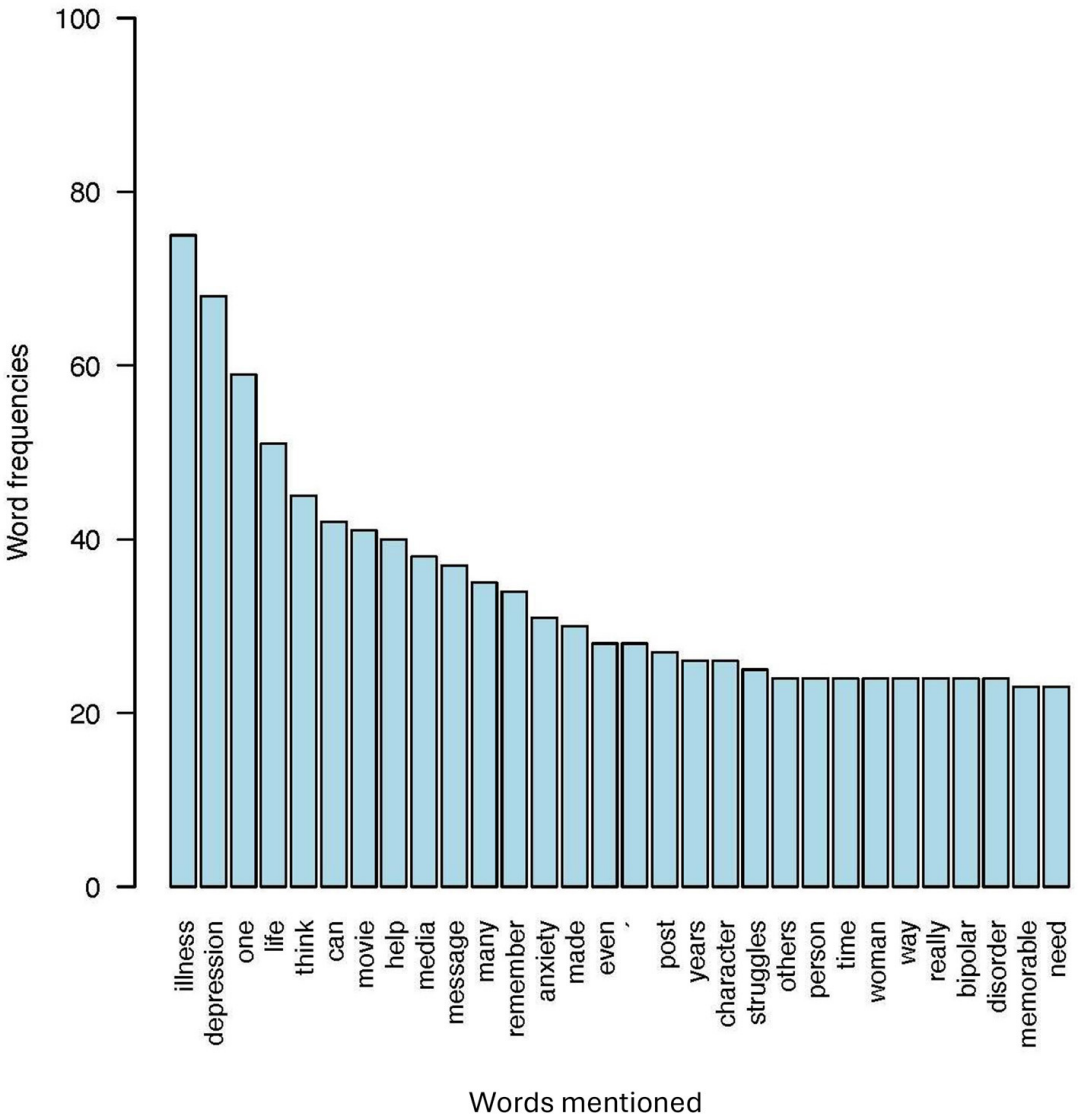
Ingroup memorable mediated MI message top word frequencies.

**Figure 6:**
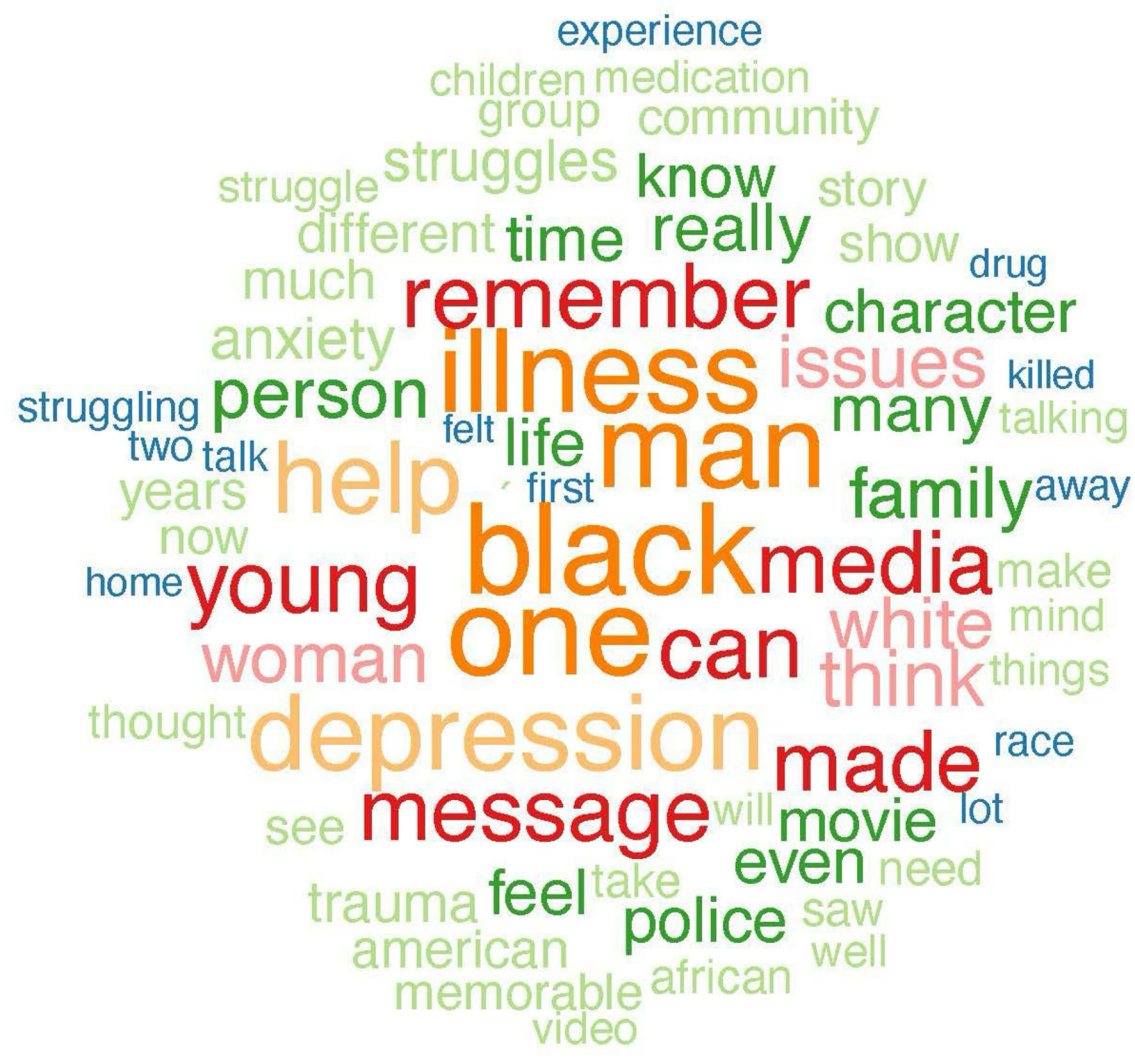
Outgroup memorable mediated MI message Word Cloud.

**Figure 7:**
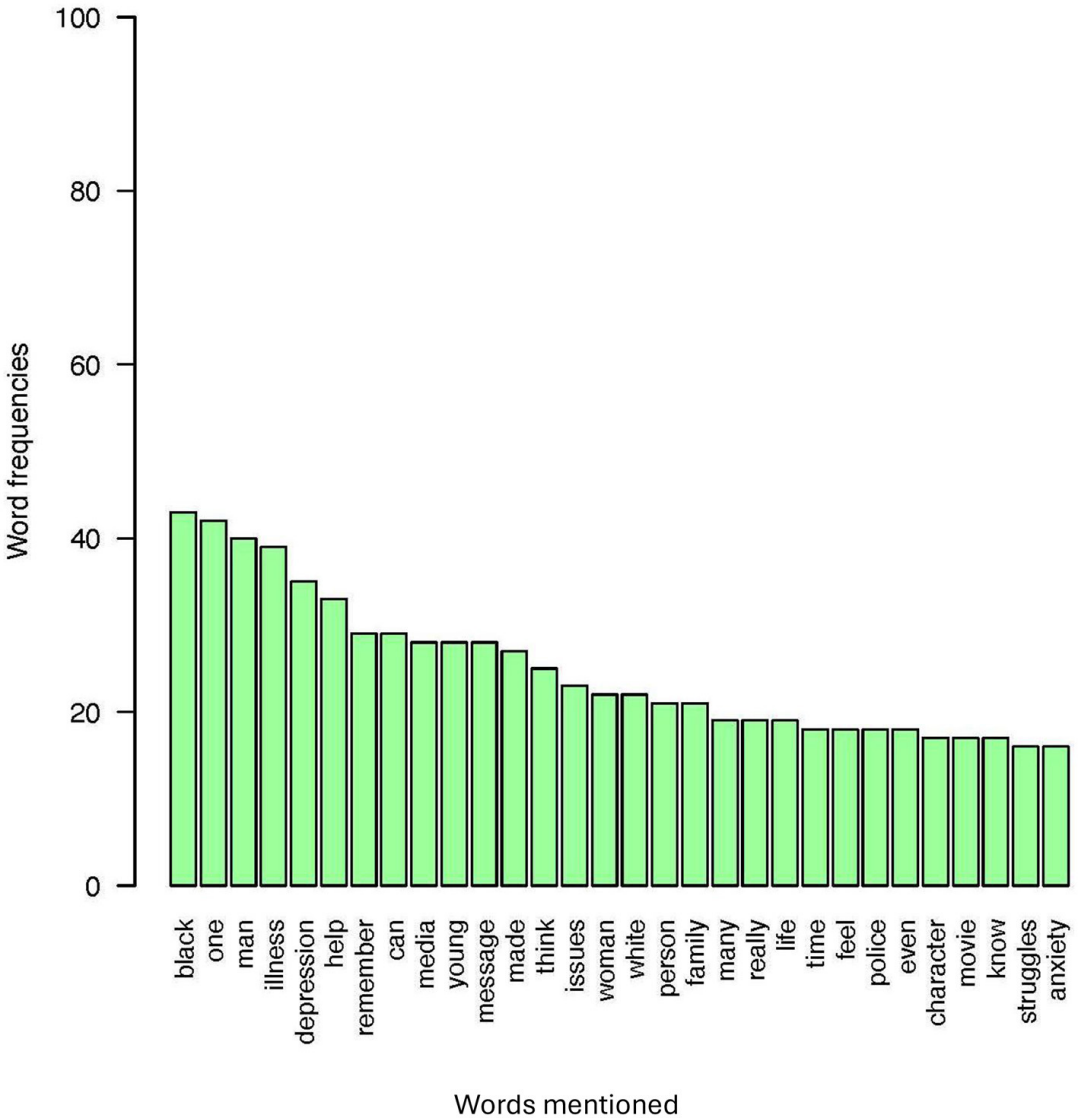
Outgroup memorable mediated MI message top word frequencies.

In reference to RQ1, examining how the content of recalled mediated memorable messages of MI differs between those who recalled racial ingroups relative to racial outgroups, the ingroup and outgroup memorable media messages about MI were compared regarding the distribution of positive, neutral, and negative language. Results of the sentiment analysis indicated that memorable ingroup messages contained a higher percentage of overall favorability than memorable outgroup messages. Specifically, 73.30% of memorable ingroup compared to 68.83% of memorable outgroup messages contained positive language. Additionally, 38.33% of memorable outgroup messages, compared to 26.70% of memorable ingroup messages, contained negative language. Only 0.83% of memorable outgroup and no memorable ingroup messages contained neutral language. Note, however, that the majority of memorable ingroup and outgroup messages contained positive language. See Table 1 for examples from the sentiment analysis. As displayed in a set of word frequency analyses represented in Figures 4–7 one qualitative difference in the top five most frequent words that were used in memorable message descriptions is the use of race in describing a MI. When recalling memories of outgroups, participants more often referenced race, notably, in the form of labeling characters as “Black.” Though no other races were mentioned to this extent in outgroup memory descriptions, no races were predominantly referenced in the ingroup condition. Similarly, gender was a top descriptor for memories involving racial outgroups in the form of describing characters as a “man.” See Figures 4, 5 for a visual word frequency representation (i.e., word cloud and bar chart, respectively) within the ingroup racial recall condition. See Figures 6, 7 for a visual word frequency representation (i.e., word cloud and bar chart, respectively) within the outgroup racial recall condition.

**Table 1:** Research question 1 sentiment analysis examples.

**Sentiment**	**Memorable message type**	**Example**
Positive	Ingroup	I saw a TV ad for a pharmaceutical treatment for depression that was very uplifting and hopeful.
Positive	Outgroup	There was a commercial for an online counseling service that was very encouraging and supportive.
Neutral	Outgroup	I recall an Italian wife whose husband had been institutionalized for mental health issues.
Negative	Ingroup	In the series Suits, Louis Litt and Harvey Specter had a big fight that was emotionally draining.
Negative	Outgroup	There was a Caucasian lady who looked depressed and it was very distressing to watch.

To explore RQ2, we examined how the content of recalled mediated memorable messages about MI may be similar between those referencing ingroups vs. outgroups. Latent Dirichlet Allocation (LDA) was used to identify common themes within each group of messages. Three ingroup and three outgroup themes emerged (see Table 2).

**Table 2:** Research question 2 thematic analysis frequencies and common words.

**Memorable message type**	**Theme**	**Frequency (*n*)**	**Common words**
Ingroup	1. Community issues, health, and media representation	30.68% (54)	Movies, issues, medication, character, African, White, community, health, message
Ingroup	2. Personal and group identity, help, and trauma	50% (88)	Medication, help, group, character, know, man, young, life, memorable, trauma
Ingroup	3. General media messages, mental health, and social interactions	19.32% (34)	Message, media, time, woman, mental, know, life, Black
Outgroup	1. Public health issues, community, wellbeing, and racial identity	17.50% (21)	Mental, health, issues, community, years, American, children, Black, illness, people
Outgroup	2. Situational stories, racial dynamics, and family	36.67% (44)	Man, Black, depression, White, help, person, woman, people, family, young
Outgroup	3. Media influence, mental health awareness, and societal perceptions	45.83% (55)	Mental, health, illness, media, message, remember, think, time

### Ingroup theme 1: community issues, health, and media

This theme reflects how approximately one-third of recalled ingroup memorable messages about MI focused on societal, health-related issues at the community level as portrayed in the media. Mental health was framed in these responses in terms of portrayed characters' positive or negative MI experiences within a group or community. Examples frequently mentioned movies, among some additional media contexts, and included references to access to mental health care, perceptions of mental illness, and portrayal of mental illness within society and communities.

### Ingroup theme 2: personal and group identity

The next theme was the most dominant, with approximately half of ingroup memorable MI media messages focusing on individual experiences and treatment of mental illness. Typically, these messages focused on one media character's mental struggle and their perceived success in improving a difficult situation. Advertisements were commonly mentioned with a particular focus on pharmaceutical commercials that depicted an individual's struggle and then their improvement due to taking a medication. In contrast to Ingroup Theme 1, this theme was less focused on society and community and more focused on the individual. However, group identity was salient in terms of participants frequently describing characteristics of the depicted characters in terms of age, gender, and other identity factors.

Although social identity characteristics were referenced, there was considerable focus on more personal experiences than community or group dynamics. The prevalence of this theme in recalled ingroup messages may suggest the salience of pharmaceutical advertisements, which are often designed to speak to the individual. This technique, as well as the strategic portrayal of diverse characters to appeal to commercial target groups, may encourage a focus on the personal rather than the collective. A positive element of these memorable messages was that participants retained information about the potential effectiveness of treatment for mental illness.

### Ingroup theme 3: general media messages, mental health, and social interactions

The final theme that emerged from the ingroup memorable messages was focused more generally on how media portrayed mental health. Multiple media sources were referenced without one particular source emerging. The most frequently mentioned social identities within this theme were “woman” and “Black,” suggesting that responses within this them were overrepresented in referencing the experiences of these groups of people. Theme responses also referenced the impact of mental health on people's lives and relationships, but not specifically in terms of treatment or society. Thus, these memorable messages reflected broader statements about the impact of mental illness on people's lives, particularly related to women and Black Americans.

### Outgroup theme 1: public health issues, community, and racial identity

This outgroup theme centered on memorable messages related to public health and communities. This was the only theme where “children” emerged as a frequent word, reflecting some salience of the idea of how families within communities are affected by mental illness. Participants recalled media messages about how MI affected others and society, not just an individual. Responses were more collective in focus, and when referencing individuals, they did so most frequently in relation to Black Americans and children.

### Outgroup theme 2: situational stories, racial dynamics, and family

In contrast to Outgroup Themes 1 and 3, this theme revealed a focus on personal stories for specific racial, gender, age, and family groups. Social identity characteristics of the media characters were frequently mentioned, with “man,” “Black,” “White,” “woman,” and “young,” making up 5 of the 10 frequent theme words. Memorable messages about depression were dominant in this theme, with descriptions of advertisement, TV, and movie characters' depressive symptoms or episodes. These messages centered on a character's individualized experience with mental illness, their social identity, and how it impacted their personal life and family.

### Outgroup theme 3: media influence, mental health awareness, and societal perceptions

The presence of this theme emerged in outgroup messages that emphasized media's influence on perceptions of mental health. These recalled messages did not have a dominant focus on specific social identity characteristics and instead focused on the impact of MI within society more generally and the media's role in imparting information about mental health. Participants suggested that the memorable message raises awareness about how MI affects groups of people and society.

This was the most commonly occurring Outgroup Theme, perhaps suggesting that when prompted to think about a memorable media message about MI that focuses on an outgroup, participants often recalled generalized messages not specific to conclusions about a particular group's experience. The social distance between the self and the outgroup may result in the focus on the media and societal perceptions rather than the personal experiences of outgroup members.

Looking across both ingroup and outgroup messages, themes overlapped in terms of discussion of mental health, media narratives and influence, and public perceptions of MI, as reflected by Ingroup Theme Three and outgroup Theme Three. Indeed, the overlap between Ingroup and Outgroup Theme Three was highlighted in the analysis. Examination of the other themes shows some additional semantic similarity, however, not as pronounced as Theme Three. For Theme One, both ingroup and outgroup memorable message responses focused on community and public health, with racial groups explicitly represented in responses for the ingroup and outgroup. Comparing Theme Two for both ingroup and outgroup memorable messages, both themes centered on individual and group narratives of mental health, however only outgroup memorable message responses referenced race (specifically, Black and White), further substantiating this aforementioned pattern observed in the semantic frequency analyses.

The LDA results suggest that both memorable ingroup and outgroup messages discuss some similar issues however with unique context and focus. These results are consistent with the word analysis conducted in R to examine frequency of word distribution across ingroup and outgroup memorable messages. The topic analysis provides further context as to how words commonly appear together and with what meaning. As displayed in a set of word frequency analyses represented in Figure 6, one qualitative similarity in the top five most frequent words that were used in memorable message descriptions is the focus on depression when describing MI media messages. With regard to both ingroup and outgroup recall, “depression” was a top five mental health condition that was explicitly mentioned. In both recall conditions, participants also were keen to talk about MI as an “illness,” employing this word far more than the other component of the health identity label (i.e., “mental”) when describing their memories.

## Discussion

A wealth of prior research has demonstrated the capacity for media exposure featuring MI to influence judgments about mental health, and those managing mental health conditions (e.g., Kresovich, 2022; Lee et al., 2021). Though it is commonly argued that such messages influence these judgments by way of the mental representations that are developed through exposure, researchers, to this point, have not sufficiently examined how particularly memorable representations could influence judgments about those managing MI, particularly judgments regarding public policy support inclinations. The current study undertook just such an investigation while examining how social identity, in the form of racial/ethnic identification, could attenuate how individuals are prone to reach broader conclusions about MI, including the necessity for allocating resources to this routinely stigmatized health identity. Additionally, the notable stigma-facilitating attribute of illness culpability was assessed as a potential explanatory mechanism to contextualize how memorable message recall of different racial/ethnic identities could prompt varied modes of support for MI initiatives more broadly. Findings reveal that culpability perceptions were an important mediator for explaining such a relationship, however, patterns of whether ingroup or outgroup recall would be precipitate more culpability for a MI condition ran counter to expectations. Implications of these findings and future directions are discussed below.

A notable pattern that was observed that run counter to hypothesized expectations was that memorable media message recall of racial outgroups was associated with less perceived culpability for a MI condition than recall of racial ingroups. Such a pattern is at odds with fundamental attribution bias principles which would suggest that greater personal culpability for stigmatized attributes would be assigned when considering outgroups and increasingly distant social others (Krull et al., 1999). It is possible that our observed finding manifested as a result of the media figure persona representing an additional salient identity intersection (in combination with the MI health identity and the racial identity), which contributed to functionally altered ways of perceiving of stigma culpability. We note here that celebrity status of the recalled message subjects was not specifically measured in the study but identified in participants' responses, referencing, for example, Simone Biles and Kanye West disclosures, amongst others. Researchers have observed that when racial minority celebrities disclose mental health concerns, this is one of the few scenarios in which stigma is not exacerbated by the combined marginalized health and racial identity (Calhoun and Gold, 2020; Cassilo, 2020). Indeed, when celebrity status is associated with individuals at this type of identity intersection, coverage and public responses will often emphasize the pronounced value of such disclosures to other minorities (Francis, 2021) who are often experiencing stress and anxiety as a result of being a marginalized racial minority (i.e., minority stress; Meyer, 2003). Given that our sample was predominantly White, it is possible that there was a recognition that racial minority status and minority stress were, in part, experiences that facilitate MI consequences in ways that individuals experiencing these outcomes would not be viewed as personally responsible.

Indeed, though instructions for both conditions primed race as a consideration, both our word frequency analysis and LDA indicated that racial considerations were primarily salient when describing memories involving outgroups. As such, sociocultural context may have been more readily employed in culpability judgments. Still, though ANOVA results reveal MI culpability ratings to be lower for racial outgroups than ingroups, LLM assessments of the content of memories revealed ingroup memories to be relatively more favorable for ingroups (i.e., proportion of positive valence descriptors was 73.30% for ingroup compared to 68.83% for outgroup, while negative language was 26.70% for ingroups and 38.33% for outgroups). These patterns suggest some degree of valence divergence with negative language, especially, relatively more assigned to racial outgroups. Furthermore, these patterns indicate that the observed favorability toward outgroups in relation to culpability judgments did not extend to outgroups, more generally. As such, culpability judgments would appear to be a unique type of perception with regard to how it runs counter to SIT predictions of ingroup favorability. Again, the historical context of race relations, notably regarding minorities in America, may attenuate how respondents think about individual responsibility for mental distress and/or trauma. This is a pattern that should be explored further in future research.

As represented in Table 2, LDA further revealed that though explicit discussions of media representation were evident in both recall of ingroups as well as outgroups, explicit discussions of the influences of recalled depictions were primarily a component of outgroup recall. Typically, media exposure is less of a potent influence on that with which individuals have direct experience (Mutz et al., 2010), which may, in part, explain why the potential for being influenced was more salient for those recalling outgroups. Participants were, on some level, demonstrating this phenomenon of direct experience with racial ingroup memorable messages of MI not explicitly being described as altering worldviews and beliefs as was more often observed to be the case for those participants recalling outgroups.

In general, even though all participants were prompted to discuss memorable media, as demonstrated in Table 2, those assigned to the ingroup condition still often discussed themes related to personal identity, whereas those discussing outgroups would contrastingly emphasize the societal influences of media and need for mental health awareness campaigns. Conceptually, this pattern, in concert with our experimental observations of influences on MI culpability perceptions, may align with notable research examining episodic relative to thematic framing on other forms of stigma culpability (e.g., poverty; Iyengar, 1991). When memorable messages of ingroups were discussed, the descriptions emphasized a relatively more individualized and episodic set of considerations. When outgroup messages were under consideration, however, thoughts more often gravitated thematically toward the role of society and broad awareness campaigns. It was also under these outgroup conditions that the least culpability was quantitatively assigned to those managing MI for their stigmatized identity, a noted implication of thematic issue considerations (Iyengar, 1991). As such, the framing with which people internalize MI management appears to differ somewhat by the identity of those recalled. In this way, such findings further support the need to give concerted attention to the identity characteristics of who is recalled within memorable messages in order to better understand the content and influences of those recalled messages.

Supporting our predictions, it was observed that as MI culpability perceptions increased, there were decreases in policy support. Such a pattern is in alignment with prior health communication research suggesting greater culpability attributions were negatively associated with intervention advocacy for those managing illness (e.g., Smith, 2012). However, due to the observed pattern of racial outgroup recall increasing perceived MI culpability, significant counter-support for our prediction that racial outgroup recall would indirectly diminish policy support intentions, via culpability, was observed. This pattern may logically also be explained by way of a potentially intersecting salience of celebrity identity with our health and racial identity nexus. If verified, such a pattern speaks to the necessity of interrogating identity in increasingly complex ways that maintain a rich awareness of the manner in which various multifaceted identity dimensions (e.g., class- and occupational-based identities) could be altering how other marginalized identities (e.g., racial- and health-based identities) are challenged or supported (Ramasubramanian and Banjo, 2020). While we acknowledge this as an informed speculation since we did not measure celebrity status specifically, future research should continue more centrally applying an intersectionality framework as it could produce a deeper and more nuanced perspective about how an individual's multiple identities influence how they are recalled and the support they receive.

Findings further revealed that, among racial outgroups, recall of Black media figures associated with MI were linked with the greatest culpability perceptions for MI, in general. These findings reflect that, when thinking about racial others, Black individuals may be on the receiving end of some of the worst MI stigma outcomes. Such observed patterns are consistent with research examining the content of racialized MI portrayals. Notably in the news landscape, evidence indicates that MI associated with minorities, particularly Black individuals, tends to be presented most often with associations of criminality and stigma (Heitzeg, 2015; Lindsay and Vuolo, 2021; Smiley and Fakunle, 2016), such as culpability attributions. Though this portrayal trend did not result in more culpability perceptions than was observed for various forms of ingroup racial recall, it still suggests that in the context of considering social others, the compounded stigmatization of Black individuals with MI management appears to most notably influence broader MI culpability perceptions. Such a pattern is all the more notable given that, according to our semantic frequency analysis, when non-Black individuals think of a memorable media message of a racial outgroup with MI, they overwhelmingly think of Black individuals.

Even though, when recalled as an outgroup, Black individual recall was associated with particularly stigmatizing perceptual attributes, when recalled as an ingroup, individuals with MI were viewed with the least culpability perceptions compared with all other conditions. This is to say, when Black respondents recalled Black media figures with MI, this was the context in which there were the most overall muted perceptions that people with MI are responsible for their condition. It would appear that Black media consumers are notably sympathetic toward MI when the intersecting identity is a racial ingroup, and that this sympathy most potently facilitates the greatest desire to support policy aiding those with MI.

Another key observation concerned the fact that White individuals assigned more culpability for MI when recalling a White media figure with MI than non-White individuals assigned when recalling a White media figure with MI. These recall conditions resulted in more favorability toward policy support for MI by the latter, relative to the former. Such patterns suggest that racial minorities are less prone to stigmatize someone with MI when they are White than White individuals are. Though somewhat unexpected, it is possible to contextualize these findings by way of frameworks like system justification theory. According to system justification theory, individuals will often “defend and justify the status quo to bolster the legitimacy of the existing social order” (Jost et al., 2004, p. 887). This tendency can result in majority domination and/or minority self-subordination. This is to say, when valuing the certainty and familiarity of the status quo, minority groups may, at times, bolster the majority's attributes and/or marginalize those related to the marginalized, including themselves. Though speculation, it is possible that, within the context of MI, minorities were less likely to ascribe inherently stigmatizing attributes to the dominant culture due to an overarching acceptance of situational explanations for mentally disordered behavior committed by members of this racial identity. White respondents may not have engaged in such attributional processing to the same extent, in part, because identity salience, and its implications, are typically not as palpable for majority group members (David et al., 2002). In the current context, White respondents may not have been as prone to protect their self-image and racial identity because, unlike racial minorities, these individuals are less likely to process experiences in the context of race. Such disparate ways of being influenced, even unconsciously, by ingroup racial identification may account for why when White individuals recalled White media figures, this was the condition in which the most stigmatizing perceptual and policy support outcomes were observed.

Despite the informative nature of emergent patterns in this study, one notable limitation revolved around the fact that, while ingroup/outgroup recall was manipulated, the race of the individual recalled for outgroups was not manipulated. This is to say, participants were allowed to consider any outgroup racial identity that was most accessible at the time of recall. Though we could have instructed participants to think of a specific racial identity, we chose not to pursue this route because of how it might have artificially confounded which memorable messages were most accessibly recalled. Indeed, many participants likely did not have a memorable mediated message of MI featuring certain racial identities. As such, the fact that White and Black outgroup recall were the most prominent is informative, in and of itself. These racial identities are, generally speaking, the most represented within the American media landscape (Dixon, 2019), which would be consistent with our findings. Even racial minorities mostly recalled White media figures when instructed to recall an outgroup. However, we recognize that the demographic makeup of the sample may still have influenced the social identity processes at play. This racial composition warrants deeper consideration, particularly with regard to the ingroup recall condition, the interpretation of ingroup/outgroup effects, and the broader generalizability of our findings. The racial homogeneity of our sample may have shaped the nature of recalled messages in ways that are not fully representative of more racially diverse populations. As such, future research would benefit from greater racial diversity to further explore how racial identity moderates the recall and interpretation of memorable media messages involving MI. Such a design objective would statistically equip researchers with a greater ability to discern patterns related to racial match or crossing between participants and their recalled messages. In the current study, such patterns were only observed and discussed related to Black and White participants and media figures within the examination of H5 because these were the racial identities most represented in the media environment and study sample.

Furthermore, within the current study, we did not assess the impact of additional identity intersections (e.g., gender, age, class) of the characters recalled. Though beyond the scope of the current investigation, it would be ostensibly quite informative to further understand how increasingly multifaceted identity intersections facilitate different ways of (de)stigmatizing MI perceptions and policy inclinations (Jackson et al., 2016). Additional research is necessary to continue exploring how the manner in which media exposure to MI is recalled can influence broader personal and social outcomes. Though not often situated within the domain of mediated communication, memorable message influence assessments hold great value for more comprehensively understanding how media use can influence support for one another. Future research must continue to explore how and why who is recalled in a given situation can facilitate potentially dramatic variations in media users' experience of social concern.

From the perspective of memorable message theorizing, our findings suggest that the social identity of the person associated with a message significantly influences not only what is remembered but also how that memory influences broader social and policy orientations. This supports and extends memorable message theorizing by indicating that the content and impact of memorable messages are not solely functions of message features or personal relevance but also of intergroup social dynamics. This study further demonstrates how exposure to information about racial outgroup members with MI activates particular mental schemas that generalize to judgments beyond the specific case, in alignment with theories of cognitive accessibility and association (e.g., priming). In the current case, outgroup recall reduced associations of personal blame for MI. This suggests that intergroup cognitive association effects may be more complicated than models articulating relatively uniform ingroup favorability (e.g., social identity theory) may suggest. Practically, these insights underscore the power of salient narrative representations to influence mental health stigma. Public health campaigns and media content creators may consider deliberately featuring diverse characters with MI, particularly from marginalized groups, to foster more widespread reductions in blame and stigma for MI, in general. Tailoring memorable, emotionally resonant messages about cultural outgroups may be a potent tool in mental health advocacy and education.

## Data Availability

The raw data supporting the conclusions of this article will be made available by the authors, without undue reservation.
